# US Medical Prices and Health Insurance Premiums, 1999-2024

**DOI:** 10.1001/jamanetworkopen.2025.47462

**Published:** 2025-12-08

**Authors:** Salpy Kanimian, Vivian Ho

**Affiliations:** 1 Department of Economics, Rice University, Houston, Texas; 2 Rice University’s Baker Institute for Public Policy, Houston, Texas; 3 Department of Medicine, Baylor College of Medicine, Houston, Texas

## Abstract

This economic evaluation describes changes in US workers’ contributions to health insurance premiums and medical costs compared with wages from 1999 to 2024.

## Introduction

For more than 160 million working people in the US and their dependents, employer-sponsored health insurance is their primary means of accessing health care.^1^ The Consumer Price Index (CPI) tracks price changes in health care services and goods affecting premiums. This study analyzes long-term trends in employer-sponsored insurance premiums and CPI over the same period to provide context for understanding premium growth and affordability challenges.^2^

## Methods

This economic evaluation was deemed exempt by the Rice University institutional review board because it is not human participants research, and follows the Consolidated Health Economic Evaluation Reporting Standards (CHEERS) reporting guideline. We conducted descriptive analyses using publicly available data. Premiums and worker contribution data were from the Kaiser Family Foundation Employer Health Benefits Survey.^3^ Inflation and wage data were from the Bureau of Labor Statistics (BLS) CPI and Current Employment Statistics Survey, covering 1999 to 2024. We constructed a longitudinal comparison of cumulative changes in 4 indicators: total family premiums for employer-sponsored insurance, mean worker contributions toward premiums, mean worker earnings, and inflation, all indexed to their 1999 values for direct comparison.

We also analyzed CPI trends in major components of medical care: professional services, hospital services, and health insurance, all normalized to 100 in January 2006, the first year BLS collected health insurance information. Professional services encompass care provided by office-based clinicians, while hospital services include both facility charges and services provided by hospital-employed physicians. The health insurance price index captures administrative expenses, retained earnings, and profits and excludes the underlying cost of care. The CPI prescription drug measure tracks retail-level price changes of medications as experienced by consumers, specifically transaction prices received by pharmacies for both prescription and prescribed over-the-counter drugs dispensed. The CPI prescription drug sample includes 350 to 400 drug molecules; it does not publicly report brand vs generic or therapeutic-class–specific indices. The drug CPI therefore reflects mean trends, instead of potentially sharp price increases for specific drugs, with details in the eMethods in Supplement 1. Data were analyzed using Stata MP version 18.5 (StataCorp) from June 12 to 15, 2025.

## Results

**Figure 1.**
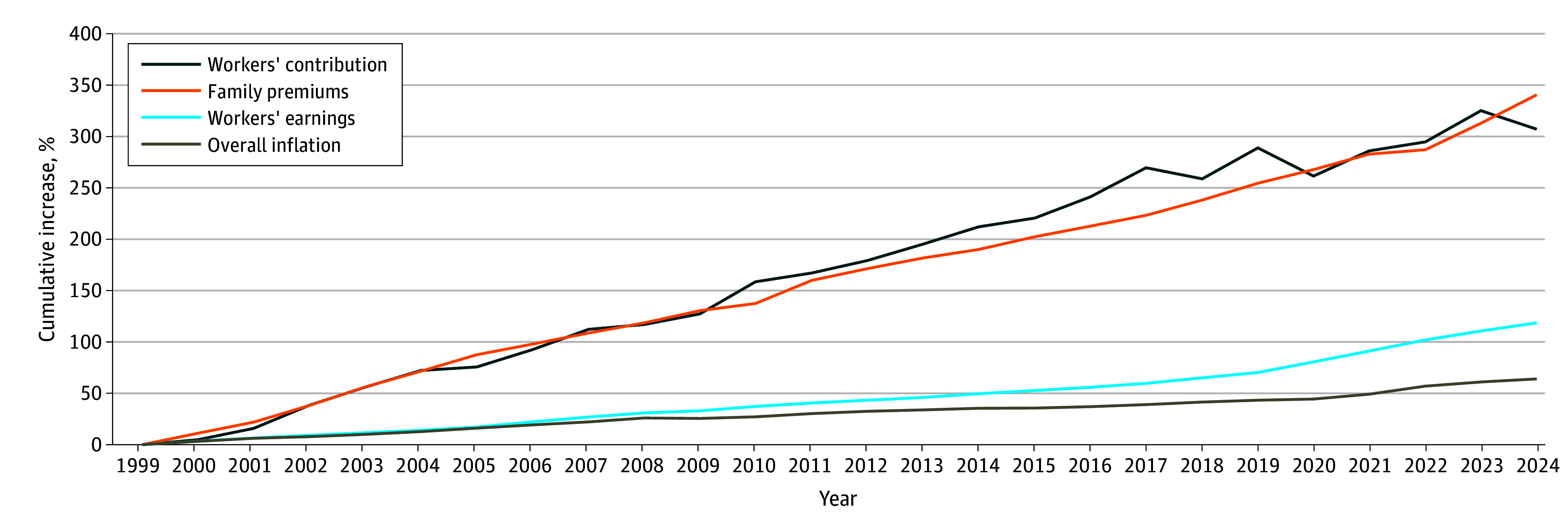
Cumulative Increases in Workers’ Contribution, Family Premiums, Overall Inflation, and Workers’ Earnings Data were collected from the Kaiser Family Foundation Employer Health Benefits Survey, 1999-2024; Bureau of Labor Statistics, Consumer Price Index, Historical Inflation Rates: 1999-2024; Bureau of Labor Statistics, Seasonally Adjusted Data from the Current Employment Statistics Survey, 1999-2024. Bureau of Labor Statistics workers’ earnings are based on the change in total mean hourly earnings of production and nonsupervisory employees. Employment, hours, and earnings data are from the Current Employment Statistics Survey by the Department of Labor.

Between 1999 and 2024, mean worker contributions toward family premiums increased by 308%, while total premiums increased by 342% (Figure 1). Over the same period, mean worker earnings increased by 119% and inflation by 64%.

**Figure 2.**
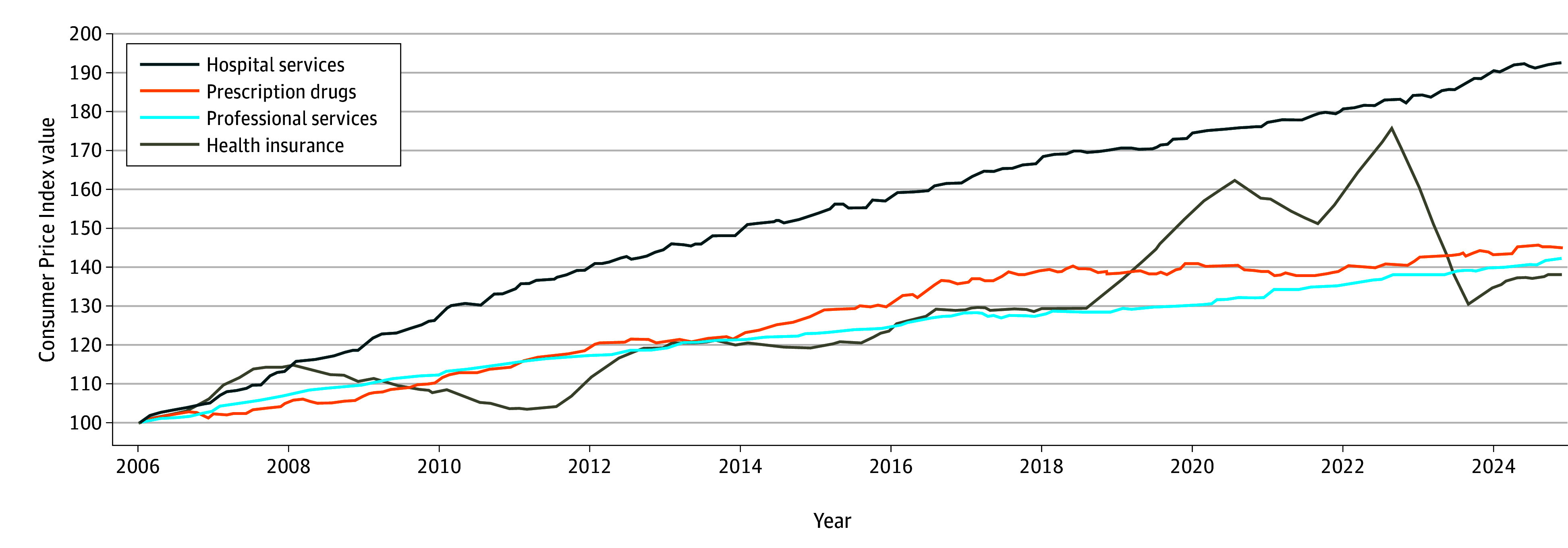
Consumer Price Index: Medical Care by Component Data were collected from the Bureau of Labor Statistics, Consumer Price Index, Medical Care Index (by Component), 2006-2024.

Figure 2 shows the trends in four components of the medical care CPI. Hospital services have increased the steepest, reaching an index value of 193 by 2024. Health insurance displayed the most volatility, peaking at 176 in 2022 then stabilizing at 138 by late 2024. Prescription drugs and professional services increased more moderately.

## Discussion

This economic evaluation found that insurance premiums have increased at 3 times the rate of workers’ earnings since 1999, accompanied by escalating hospital prices. Health insurance prices increased at rates close to hospital prices during the COVID-19 pandemic but have since stabilized. This volatility reflects both pandemic-era shifts in health care utilization (eg, limited clinician visits) and higher retained earnings for insurers. As utilization normalized and CPI updates took effect, the index dropped.

While the prescription drug CPI tracks mean price changes, it may underrepresent costs faced by consumers with chronic conditions, because the probability that a drug is included in the index is proportional to its share of total pharmacy revenues. For example, some high-cost drugs have seen extreme price hikes, with nearly 300% price increases since launch.^4^

Hospital prices may be the primary cost driver, even as per capita admissions (except for outpatient visits) have declined.^5^ Given the descriptive nature of this analysis, we cannot draw inference-based conclusions about whether changes in specific CPI components are driving premium increases. The findings should be interpreted as contextual trends rather than evidence of causal relationships, with the additional caveat that price measurement in a rapidly innovating sector remains challenging.

## Notes

Supplement 1.
eMethods.

Supplement 2.
Data Sharing Statement
