# Supplementary material for: US Medical Prices and Health Insurance Premiums, 1999-2024
Source: JAMA Netw Open. 2025 Dec 8;8(12):e2547462. doi: 10.1001/jamanetworkopen.2025.47462 (PMC12687089; doi:10.1001/jamanetworkopen.2025.47462)
Supplement: Supplement 1. — eMethods. [file jamanetwopen-e2547462-s001.pdf]

Supplemental Online Content

Kanimian S, Ho V. US medical prices and health insurance premiums, 1999-2024. *JAMA Netw Open*. 2025;8(12):e2547462. doi:10.1001/jamanetworkopen.2025.47462

eMethods

This supplemental material has been provided by the authors to give readers additional information about their work.

A. Worker's Earnings, Premium Contributions, Family Premium, and Inflation

To track employer-sponsored coverage premiums (Figure 1), we use data from two main sources. We use KFF Employer Health Benefits Survey for Premiums and Worker Contributions Among Workers Covered by Employer-Sponsored Coverage (family), 1999-2024.[1,2] Since 1999, the Employer Health Benefits Survey has surveyed on a yearly basis private and non-federal public employers with three or more employees to document trends in employer-sponsored health insurance such as premium of their health coverage, worker contributions, etc. We compare the trends in employer-sponsored health insurance to overall inflation and worker's earnings using data from the Bureau of Labor Statistics (BLS). BLS's Worker's earnings is based on the change in total average hourly earnings of production and nonsupervisory employees. Employment, hours, and earnings data is from the Current Employment Statistics Survey by the Department of Labor.[3] Annual Inflation data are from the BLS's Consumer Price Index (CPI) historical inflation rates: 2000-2024.[4]

We calculate the cumulative percentage increases in each of the following variables: (1) worker contributions, (2) premiums, (3) worker earnings, and (4) overall inflation starting with 0% in 1999 until 2024 using the following formula:

$$Cumulative\ \%\ increase\ in\ year\ t = \frac{[Variable\ in\ year\ t\ -\ Variable\ in\ year\ 1999]}{Variable\ in\ year\ 1999} * 100$$

B. Consumer Price Index: Hospital Services, Physician Services, Health Insurance, and Prescription Drugs

For the drivers behind rising employer-sponsored insurance costs, we analyze trends in CPI for the major components of medical care during 2006-2024. BLS's medical care index is composed of medical care services and medical care commodities. The medical care services is the larger component in terms of weight in the CPI, calculated by tracking consumer out-of-pocket expenses (patient direct payments for medical goods and services and premium), and divided into professional services, hospital and related services, and health insurance.

The professional services index covers services that are performed and billed by private-practice medical doctors, dentists, eye care providers, and other medical providers. The pricing unit is a doctor's visit, defined by a specific medical service. At the initial visit, BLS establishes the practitioner's specialty; if it is a group practice, one practitioner is sampled. Then a medical service is sampled via PPS. For Physicians' Services, Current Procedural Terminology (CPT) codes are collected to help describe the item accurately.

The hospital services index tracks the price of services (both inpatient and outpatient) performed and billed by a hospital or a physician employed by the hospital. The pricing unit is a hospital visit, defined by a specific medical service and diagnosis. At the initial visit, BLS works with the respondent to select a medical service using a process known as sampling by probability proportional to size (PPS). They then document the medical service and specific procedures of the hospital visit.

The health insurance index covers health insurance premiums. Since it is challenging to control for changes in quality such as policy benefits and risk factors, BLS uses the retained earning approach to calculate this index. Health Insurance index calculation was subject to many changes in terms of data, but in all cases, retained earnings were used, except for a change announced in October 2023. The retained earnings ratio used in the health insurance index is lagged on average by 10 months. We account for this lag before generating Figure 2. Notably, the health insurance CPI is calculated with a 10-month lag and reflects changes in retained earnings rather than direct changes in premium prices. Note that only the retained earnings ratio for health insurance is lagged. The October 2022 CPI represents 2021 annual retained earnings. For the other components, the October 2022 CPI reflects October 2022 prices.

Prescription drug CPI tracks retail-level price changes of medications as experienced by consumers. The drug sample is regularly updated: retail outlets and drugs are re-sampled twice a year, with each item typically priced for

about 4 years. Sampling is based on recent sales data: pharmacies report the last 20 dispensed prescriptions, and drugs are selected with probabilities proportional to their revenue share. If a drug goes off-patent, a 6-month window is used to reallocate sampling between brand and generic versions based on dispensed volumes.

We normalize January 2006 CPI to 100 for all components and adjust changes accordingly to match changes in the health insurance price index. That is, for all components, $new\ CPI_{January\ 2006} = 100$

$$new\ CPI\_t = new\ CPI_{t-1} + 100 * \frac{old\ CPI_{t-1} - old\ CPI_t}{old\ CPI_t}$$ where t is month

C. *Physician Visits and Prescription Drug Utilization*

We use Medical Panel Expenditure Survey (MEPS) Public Use Files (PUF) via Integrated Public Use Microdata Series (IPUMS). MEPS collects data on health care use, health care expenses, health insurance coverage, and quality of care, among other related topics. For physician visits, we use the variable OBPHVIS, which captures the total number of visits to an office-based physician during the year.[11] For prescription drug utilization, we use the variable RXPRMEDSNO, which is the count of all prescribed medications purchased during the year (including initial purchases and refills).[12]

We calculate the annual average number of physician visits and prescription medications purchased as measures of utilization using the following formula:

$$\bar{V}_t = \frac{\sum_{i=1}^{N} w_{it} v_{it}}{\sum_{i=1}^{N} w_{it}}$$

where i index individuals, *i=1,…,N* in MEPS. w_{it} is person weight for individual *i* in year *t*. For average per capita annual physician visits, v_{it} is the number of office-based physician visits, and for the prescription drugs purchased, v_{it} is the number of prescription medications purchased for individual *i* during the year *t.*

References

1. Family Premiums: KFF Employer Health Benefits Survey, 2024

https://www.kff.org/interactive/premiums-and-worker-contributions-among-workers-covered-by-employer-sponsored-coverage/

2. Worker's (Family) Contribution: KFF Employer Health Benefits Survey, 2024. https://www.kff.org/interactive/premiums-and-worker-contributions-among-workers-covered-by-employer-sponsored-coverage/
3. Worker's Earnings: Bureau of Labor Statistics and based on the change in total average hourly earnings of production and nonsupervisory employees. Employment, hours, and earnings from the Current Employment Statistics survey, Department of Labor. https://beta.bls.gov/dataViewer/view/timeseries/CES0500000030
4. Annual Inflation: Historical Inflation Rates: 2000-2024, Consumer Price Index, Bureau of Labor Statistics. https://www.usinflationcalculator.com/inflation/historical-inflation-rates/
5. Consumer Price Index for All Urban Consumers: Medical Care in U.S. City Average not seasonally adjusted [CUUR0000SAM], retrieved January 6, 2025. https://data.bls.gov/dataViewer/view/timeseries/CUUR0000SAM
6. Consumer Price Index for All Urban Consumers: Professional Services in U.S. City Average not seasonally adjusted [CUUR0000SEMC], retrieved January 6, 2025. https://data.bls.gov/dataViewer/view/timeseries/CUUR0000SEMC
7. Consumer Price Index for All Urban Consumers: Hospital and Related Services in U.S. City Average not seasonally adjusted [CUUR0000SEMD], retrieved January 6, 2025. https://data.bls.gov/dataViewer/view/timeseries/CUUR0000SEMD
8. Consumer Price Index for All Urban Consumers: Hospital Services in U.S. City Average not seasonally adjusted [CUUR0000SEMD01], retrieved January 6, 2025. https://data.bls.gov/dataViewer/view/timeseries/CUUR0000SEMD01
9. Consumer Price Index for All Urban Consumers: Health Insurance in U.S. City Average not seasonally adjusted [CUUR0000SEME], retrieved January 6, 2025. https://data.bls.gov/dataViewer/view/timeseries/CUUR0000SEME
10. Consumer Price Index for All Urban Consumers: Prescription Drugs in U.S. City Average not seasonally adjusted [CUUR0000SEMF01], retrieved January 6, 2025. https://data.bls.gov/dataViewer/view/timeseries/CUUR0000SEMF01
11. Medical Panel Expenditure Survey, Office-Based Providers, retrieved June 12, 2025. https://meps.ipums.org/meps-action/variables/OBPHVIS#description_section
12. Medical Panel Expenditure Survey, Collection of Information About Prescription Medications, retrieved June 12, 2025. https://meps.ipums.org/meps/userNotes_RX_medications.shtml
