# Supplementary material for: US Medical Prices and Health Insurance Premiums, 1999-2024
Source: JAMA Netw Open. 2025 Dec 8;8(12):e2547462. doi: 10.1001/jamanetworkopen.2025.47462 (PMC12687089; doi:10.1001/jamanetworkopen.2025.47462)
Supplement: Supplement 2. — Data Sharing Statement [file jamanetwopen-e2547462-s002.pdf]

Data Sharing Statement

Kanimian. US Medical Prices and Health Insurance Premiums, 1999-2024. *JAMA Netw Open*. Published December 08, 2025. doi:10.1001/jamanetworkopen.2025.47462

Data
Data available: Yes
Data types: Data (not involving human participants), Data dictionary
How to access data: All data requests should be sent to corresponding author, Salpy Kanimian, at salpy@rice.edu
When available: With publication

Supporting Documents
Document types: Statistical/analytic code
How to access documents: All data requests should be sent to corresponding author, Salpy Kanimian, at salpy@rice.edu
When available: With publication

Additional Information
Who can access the data: All those who request.
Types of analyses: For any purpose.
Mechanisms of data availability: Any.
Any additional restrictions: All data requests should be sent to corresponding author, Salpy Kanimian, at salpy@rice.edu
